# Multi-omics analyses demonstrate the modulating role of gut microbiota on the associations of unbalanced dietary intake with gastrointestinal symptoms in children with autism spectrum disorder

**DOI:** 10.1080/19490976.2023.2281350

**Published:** 2023-11-27

**Authors:** Hailin Li, Churui Liu, Saijun Huang, Xin Wang, Muqing Cao, Tingfeng Gu, Xiaoxuan Ou, Shuolin Pan, Zongyu Lin, Xiaotong Wang, Yanna Zhu, Jin Jing

**Affiliations:** a Department of Maternal and Child Health, School of Public Health, Sun Yat-sen University, Guangzhou, Guangdong, China; b School of Biological Engineering, Dalian Polytechnic University, Dalian, Liaoning, China; c Department of Child Healthcare, Affiliated Foshan Maternity and Child Healthcare Hospital, Southern Medical University, Foshan, Guangdong, China; d Key Laboratory of Brain, Cognition and Education Science, Ministry of Education, Institute for Brain Research and Rehabilitation, and Guangdong Key Laboratory of Mental Health and Cognitive Science, South China Normal University, Guangzhou, Guangdong, China

**Keywords:** Unbalanced dietary intake, gut microbiota, metabolomics, gastrointestinal symptoms, autism spectrum disorder

## Abstract

Our previous work revealed that unbalanced dietary intake was an important independent factor associated with constipation and gastrointestinal (GI) symptoms in children with autism spectrum disorder (ASD). Growing evidence has shown the alterations in the gut microbiota and gut microbiota-derived metabolites in ASD. However, how the altered microbiota might affect the associations between unbalanced diets and GI symptoms in ASD remains unknown. We analyzed microbiome and metabolomics data in 90 ASD and 90 typically developing (TD) children based on 16S rRNA and untargeted metabolomics, together with dietary intake and GI symptoms assessment. We found that there existed 11 altered gut microbiota (FDR-corrected P-value <0.05) and 397 altered metabolites (P-value <0.05) in children with ASD compared with TD children. Among the 11 altered microbiota, the *Turicibacter*, *Coprococcus 1*, and *Lachnospiraceae FCS020 group* were positively correlated with constipation (FDR-corrected P-value <0.25). The Eggerthellaceae was positively correlated with total GI symptoms (FDR-corrected P-value <0.25). More importantly, three increased microbiota including *Turicibacter*, *Coprococcus 1*, and Eggerthellaceae positively modulated the associations of unbalanced dietary intake with constipation and total GI symptoms, and the decreased *Clostridium sp. BR31* negatively modulated their associations in ASD children (P-value <0.05). Together, the altered microbiota strengthens the relationship between unbalanced dietary intake and GI symptoms. Among the altered metabolites, ten metabolites derived from microbiota (*Turicibacter*, *Coprococcus 1*, Eggerthellaceae, and *Clostridium sp. BR31*) were screened out, enriched in eight metabolic pathways, and were identified to correlate with constipation and total GI symptoms in ASD children (FDR-corrected P-value <0.25). These metabolomics findings further support the modulating role of gut microbiota on the associations of unbalanced dietary intake with GI symptoms. Collectively, our research provides insights into the relationship between diet, the gut microbiota, and GI symptoms in children with ASD.

## Introduction

Gastrointestinal (GI) symptoms, one of the prevalent co-occurring conditions in autism spectrum disorder (ASD),^1^ including constipation, diarrhea, flatulence, and abdominal pain, may contribute to the pathogenesis or greater ASD symptom severity^2^ and correlate with the various aberrant behaviors (stereotypy, social withdrawal, irritability, self-injury, and hyperactivity),^3^ immune dysregulation, and metabolic dysfunction.^4^ However, the link between autism and GI symptoms is unclear and unconvincing, as not all autistic children have GI complications.^5^ GI symptoms were first associated with ASD through the presentation of feeding problems in affected children.^6^ Children with ASD are up to five times more likely than typically developing (TD) children to have feeding problems.^7^ Although a study has raised that dietary intake was not associated with GI symptoms in children with ASD.^8^ Our latest findings indicated that unbalanced dietary intake was an important independent factor associated with GI symptoms in children with ASD. It has been reviewed that diet plays a role in the development of many human GI diseases,^9^ which is consistent with our findings, supporting an association of an unbalanced diet with GI symptoms.

The microbiome-gut-brain axis has been implicated as a potential way to affect the symptoms and behaviors of ASD.^10^ These prompted researchers to examine the gut microbial composition of ASD children. Lines of studies have demonstrated that autistic children exhibited dysbiosis or altered microbial abundance.^11–13^ However, the type of altered gut microbiota and metabolites found in different studies varies widely.^14,15^ The causes of this variability between studies include, but are not limited to the substantial variability in baseline microbiome compositions across individuals, geographical regions,^16^ complexity and heterogeneity of ASD,^17^ differences in sample sizes, methodology, and covariates controlled.^18,19^ These variations contribute to the controversial conclusions regarding the association between the microbiome-gut-brain axis and ASD symptoms and behaviors. Consequently, further research with larger sample sizes, carefully controlling for covariates is required to thoroughly elucidate the gut microbiome and metabolomics features in ASD.

Diet can rapidly and reproducibly change the composition and homeostasis of microbiota.^9,20^ Berding et al. documented that an eating pattern characterized by a higher intake of healthy foods such as fruit, vegetables, legumes, nuts, and seeds was associated with a bacterial profile that could potentially be linked to some aspects of GI health.^21^ Children with ASD are mostly picky eaters and consume less healthy diets,^22^ often lacking in certain key nutrients,^8^ which unavoidably influences the composition and/or richness of the gut microbiota, leading to dysbiosis. A large autism stool metagenomics study concluded that a less-diverse diet that relates to autism diagnostic features reduced microbial taxonomic diversity and looser stool consistency.^23^ Valenzuela et al. reported that an unbalanced diet (low numbers of fruits and vegetables or foods rich in fiber) seems to have an essential role in the appearance of GI symptoms for ASD,^24^ paving the way for the proposed link between ASD-related diet, microbiome profile, and GI problems. It has been suggested that the diet-induced host phenotypes are mediated by changes in gut microbiota.^25^ Koeth et al. noted that intestinal microbiota potentially modulates the link between high levels of red meat consumption and increased atherosclerosis risk.^26^ This leads to the hypothesis that altered gut microbiota can modulate the relationship between ASD-associated unbalanced diet and GI symptoms.

Gut microbiota dysbiosis can promote GI problems through multiple mechanisms.^27,28^ Gut microbiota can produce a variety of metabolites, such as bile acids, short-chain fatty acids (SCFAs), tryptophan metabolites, and methane, which have essential effects on intestinal motility and secretion.^29^ Thus, we speculated that the altered metabolites produced by the altered microbiota might be the critical drivers of GI symptoms for children with ASD. As Snelson and his colleague noticed, the intestinal microbiota ferments indigestible carbohydrates in the diet to produce SCFAs,^30^ which regulate immune function, colonic homeostasis, and health.^31^ Similar phenomena have been observed that the intestinal microbiota may promote the development of atherosclerosis [a chronic inflammatory disorder of the large and medium-sized arterial wall and is a major underlying cause of cardiovascular disease (CVD)]^32^, through the generation of pro-atherosclerotic metabolites.^33^

Therefore, there are two main aims of this study. Firstly, we compared the microbiome and metabolomics data between the children with ASD and TD children. Secondly, we explored whether the altered gut microbiota modulates the associations of unbalanced dietary intake with GI symptoms in children with ASD via the multi-omics data analyses.

## Result

### Demographic characteristics

A total of 90 children with ASD [median age 6.1 years, interquartile range (IQR) 4.7–7.0; sex, male: female 74:16] and 90 TD children (median age 6.4 years, IQR 3.1–7.4; sex, male: female 73:17) were enrolled into this study. There were statistically significant differences between children with ASD and TD children in the demographic characteristics, including intellectual functioning, birth mode, average daily sleep duration, maternal educational attainment, and monthly per-capita income (P-value <0.05, effect size > 0.1), see Table 1.

**Table 1.** Demographic characteristics of the children with ASD and age-matched TD children.

Characteristics	ASD (*n* = 90)	TD (*n* = 90)	P-value	Effect size
**Child characteristics**				
Age, median (IQR), y	6.1 (4.7, 7.0)	6.4 (3.1, 7.4)	0.839	0.015
Sex, n (n% of male)	74 (82.2)	73 (81.1)	0.847	0.014
BMI, median (IQR), kg/m^2^	15.4 (14.4, 16.5)	15.4 (14.6, 16.5)	0.464	0.055
Age at diagnosis, median (IQR), y	3.2 (2.5, 4.3)	NA	NA	NA
Early intervention before the age of 5, n (%)	78 (86.7)	NA	NA	NA
ASD symptom severity^a^, n (%)				
Mild-to-moderate	72 (80.0)	NA	NA	NA
Severe	18 (20.0)	NA	NA	NA
Intellectual functioning^b^, n (%)				
Normal	38 (42.2)	77 (85.6)	<0.001	0.347
Borderline	9 (10.0)	8 (8.9)		
Abnormal	43 (47.8)	5 (5.6)		
Premature birth, n (%)	6 (6.7)	2 (2.2)	0.278	0.081
Birth mode, n (%)				
Vaginal delivery	53 (58.9)	57 (63.3)	0.007	0.166
Elective caeserean section	17 (18.9)	27 (30.0)		
Emergency caeserean section	20 (22.2)	6 (6.7)		
Birth order, n (%)				
1	56 (62.2)	67 (74.4)	0.078	0.131
>1	34 (37.8)	23 (25.6)		
Feeding patterns before 2 years of age^c^, n (%)				
Breastfeeding	23 (25.6)	20 (22.2)	0.871	0.028
Mixed feeding	21 (23.3)	22 (24.4)		
Artificial feeding	46 (51.1)	48 (53.3)		
Food allergy, n (%)	16 (17.8)	13 (14.4)	0.543	0.045
Antibiotic exposure before the age of 5 years^d^, n (%)	58 (64.4)	51 (56.7)	0.286	0.080
Sleep duration, median (IQR), hours/day	9.8 (9.1, 10.6)	10.3 (9.5, 11.1)	0.007	0.202
SB, median (IQR), hours/d	2.7 (1.3, 4.4)	3.0 (1.0, 5.4)	0.436	0.058
MVPA, median (IQR), hours/d	0.7 (0.4, 1.1)	0.7 (0.3, 1.2)	0.879	0.011
Walking, median (IQR), hours/d	0.5 (0.3, 1.0)	0.4 (0.2, 1.0)	0.448	0.057
**Maternal Characteristics**				
Advanced maternal age, n (%)	5 (5.6)	9 (10.0)	0.266	0.083
Educational attainment, n (%)				
College degree or below	48 (53.3)	26 (28.9)	0.001	0.248
Undergraduate degree or higher	42 (46.7)	64 (71.1)		
Gestational diabetes mellitus, n (%)	14 (15.6)	8 (8.9)	0.172	0.102
Maternal obesity, n (%)	3 (3.3)	2 (2.2)	1.000	0.000
GI symptoms during pregnancy, n (%)	21 (23.3)	17 (18.9)	0.465	0.055
Antibiotic exposure during pregnancy, n (%)	3 (3.3)	1 (1.1)	0.621	0.038
**Family characteristics**				
Monthly per-capita income, n (%)				
≤8,000 RMB	65 (72.2)	45 (50.0)	0.002	0.228
>8,000 RMB	25 (27.8)	45 (50.0)		
Parenting behavior^e^, n (%)				
Support/engagement	82 (91.1)	84 (93.3)	0.578	0.042
Opposition/defiance	8 (8.9)	6 (6.7)		

^a^Evaluated by the Childhood Autism Rating Scale (CARS). ^b^ Evaluated by the Wechsler Intelligence Scale for Children Fourth Edition (WISC-IV) or Gesell developmental Schedules (GDS). ^c^ Longest sustained feeding patterns before 2 years of age; ^d^ Children have taken antibiotics or antifungal medications before the age of 5 years. ^e^ Evaluated by the Parent Behavior Inventory (PBI). Effect size = Z/√(N) for Mann – Whitney U-tests, and effect size φ or Cramer-V for *χ*^*2*^ tests. ASD, autism spectrum disorder; TD, typically developing; IQR, interquartile range; BMI, body mass index; SB, sedentary behavior; MVPA, moderate-to-vigorous physical activity; GI, gastrointestinal; RMB, Ren Min Bi (Chinese currency).

### Altered gut microbiota in children with ASD based on the 16S ribosomal RNA (16S rRNA) data

For the rarefaction curve of the microbial richness, the X axis illustrates the Rarefaction Percentage (%), and the Y axis demonstrates the number of sequences per sample (Supplementary Figure S1). When the curves approached saturation, a reasonable sequencing depth was acquired for the investigation of the stool microbiota. For alpha diversity, there were no significant differences in Chao1, Shannon, and Simpson indexes at phylum (Wilcoxon rank-sum test, FDR corrected P-value = 0.174, FDR corrected P-value = 0.654, FDR corrected P-value = 0.828) and genus (Wilcoxon rank-sum test, FDR corrected P-value = 0.955, FDR corrected P-value = 0.391, FDR corrected P-value = 0.548) levels between children with ASD and TD children (Figure 2a-f). For microbiota beta diversity, there existed significant differences in the principal coordinates analysis (PCoA) [permutation multivariate analysis of variance (PERMANOVA) tests, P-value = 0.034] and non-metric multidimensional scaling (NMDS) (PERMANOVA tests, P-value = 0.036) based on Bray-Curtis distance (Figure 2g,h). Also, there existed significant differences in the PCoA analysis based on the unweighted Unifrac distance (PERMANOVA tests, P-value <0.001) and weighted Unifrac distance (PERMANOVA tests, P-value = 0.006) (Figure 2i,j). To document co-occurrence and interaction patterns in microbial communities, the constructed network analysis exhibited that the number of significantly (−0.4 > *r* > 0.4, FDR corrected P-value <0.001) correlated gut microbiota was strikingly higher in the TD group than that in the ASD group (Figure 2k,l). Next, the relative abundance of gut microbiota at family, genus, and species levels was compared between children with ASD and TD children. The results using both Wilcoxon rank-sum and Analysis of Compositions of Microbiomes with Bias Correction (ANCOM-BC) tests showed that the significantly increased taxa were the *Turicibacter*, *Coprococcus 1*, *Lachnospiraceae FCS020 group*, *Allobaculum*, Eggerthellaceae, Coriobacteriales Incertae Sedis, and *Megasphaera sp. BS-4* (FDR corrected P-value <0.05 and 0.25, respectively) (Figure 2m,n Supplementary Table S2). The significantly decreased taxa were the *Ruminiclostridium 6*, *Actinomycetales*, *Tyzzerella*, and *Clostridium sp. BR31* (FDR corrected P-value <0.05 and 0.25, respectively) (Figure 2m,n Supplementary Table S2). The increased or decreased taxa aforesaid belong to the Firmicutes and Actinobacteria. Moreover, the linear discriminant analysis (LDA) effect size (LEfSe) displayed that the ASD children were characterized by higher levels of Firmicutes and Fusobacteria and the TD children were characterized by higher levels of Actinobacteria and Proteobacteria (Figure 2o). The random forest analysis also identified that the Firmicutes and Actinobacteria were the main differentially abundant taxa between children with ASD and TD children (Supplementary Figure S2).

**Figure 1.**
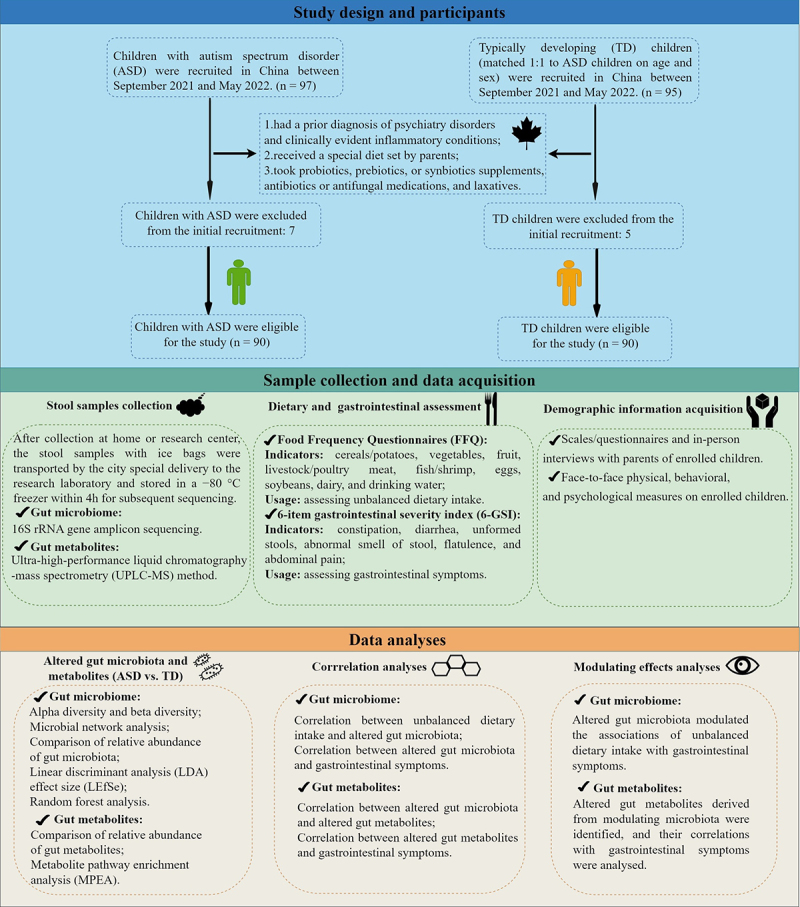
Experimental strategy for the study.

**Figure 2.**
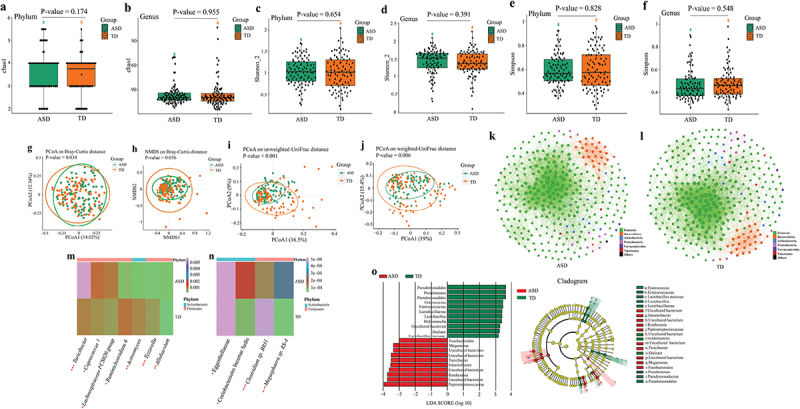
Altered gut microbiota in children with ASD based on the 16S rRNA data (*n* = 180). (a, b) The Chao1 index between children with ASD and TD children at phylum (a) and genus (b) levels. (c, d) The Shannon index between children with ASD and TD children at phylum (c) and genus (d) levels. (e, f) The Simpson index between children with ASD and TD children at phylum (e) and genus (f) levels. Statistically significant FDR correction P-value <0.05. (g, h) Principal coordinates analysis (PCoA) (g) and non-metric multidimensional scaling (NMDS) (h) of the microbiota based on Bray-Curtis distance. (i, j) PCoA analysis based on the unweighted Unifrac distance (i) and weighted Unifrac distance (j). Statistically significant P-value <0.05. (k, l) Correlation network analysis of gut microbiota in children with ASD (k) and TD children (l). The network analysis was conducted based on the Spearman correlation algorithms. Each node presents one operational taxonomic unit (OTU), and the node size indicates the relative abundance of each OTU. The straight line represents a significant correlation (−0.4 > *r* > 0.4, FDR-corrected P-value <0.001) between two nodes. (m, n) The significantly differential relative abundance of microbiota between children with ASD and TD children were tested using a Wilcoxon rank-sum test at genus level (m), at family and species levels (n). * FDR-corrected P-value <0.05, ** FDR-corrected P-value <0.01, *** FDR-corrected P-value <0.001. (o) Linear discriminant analysis (LDA) effect size (LEfSe) analysis between children with ASD and TD children. LDA scores showed differentially abundant bacterial taxa between children with ASD and TD children (LDA >3.0). Cladograms generated by LEfSe indicated differences in the bacterial taxa between children with ASD and TD children. Red bras represent taxa enrichment in ASD, and green bars indicate taxa enrichment in TD, P-value <0.05. ASD, autism spectrum disorder; TD, typically developing; 16S ribosomal RNA, 16S rRNA.

#### Altered gut metabolites in children with ASD based on the ultra-high-performance liquid chromatography-mass spectrometry (UPLC-MS) method

**Figure 3.**
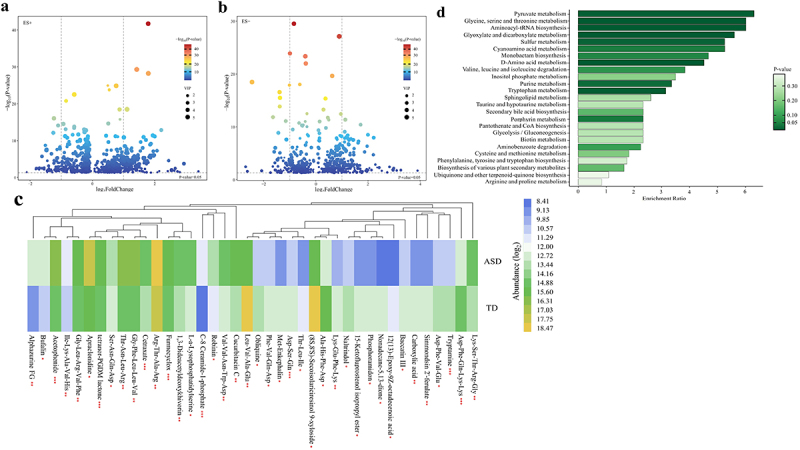
Altered gut metabolites in children with ASD based on the UPLC-MS method (*n* = 180). (a, b) Volcano plot of the significantly differential gut metabolites in the positive (a) and negative (b) ion modes. The differential metabolites were screened out according to the variable importance in projection (VIP) ≥ 1 and P-value <0.05. Two vertical lines indicate metabolites log_2_foldchange −1 and 1, respectively, and the horizontal line indicates the P-value of 0.05. The color of the dot represents the P-value levels. (c) Heatmap of the log2-transformed relative abundance of the top 40 significantly differential gut metabolites (fold change ≤ 0.5 or ≥ 2, VIP ≥ 1, and P-value <0.05). * P-value <0.05, ** P-value <0.01, *** P-value <0.001. (d) Horizontal bar charts of KEGG pathways enrichment analyses. The pathways were screened according to the significantly differential metabolites (fold change ≥1.1 or ≤0.9, VIP ≥ 1.25, and P-value <0.05). The bar lengths indicate the enrichment ratio levels of the pathways. The color of the bar represents the P-value levels. ASD, autism spectrum disorder; UPLC-MS, ultra-high-performance liquid chromatography-mass spectrometry.

Volcano plots summarized the results of significantly differential metabolites [variable importance in projection (VIP) ≥ 1 and P-value <0.05] (Figure 3a,b). Besides, the relative abundance of the top 40 significantly altered gut metabolites (fold change ≤ 0.5 or ≥ 2, VIP ≥ 1, and P-value <0.05) were displayed by the heatmap (Figure 3c). To identify important KEGG pathways with significantly altered metabolites, metabolite pathway enrichment analysis (MPEA) was carried out based on the 397 metabolites (fold change ≥ 1.1 or ≤ 0.9, VIP ≥ 1.25, and P-value <0.05) (Supplementary Table S9), finding out the 25 key metabolic pathways, further suggesting a different metabolic state (Figure 3d).

### The correlation between unbalanced dietary intake and altered gut microbiota in children with ASD

**Figure 4.**
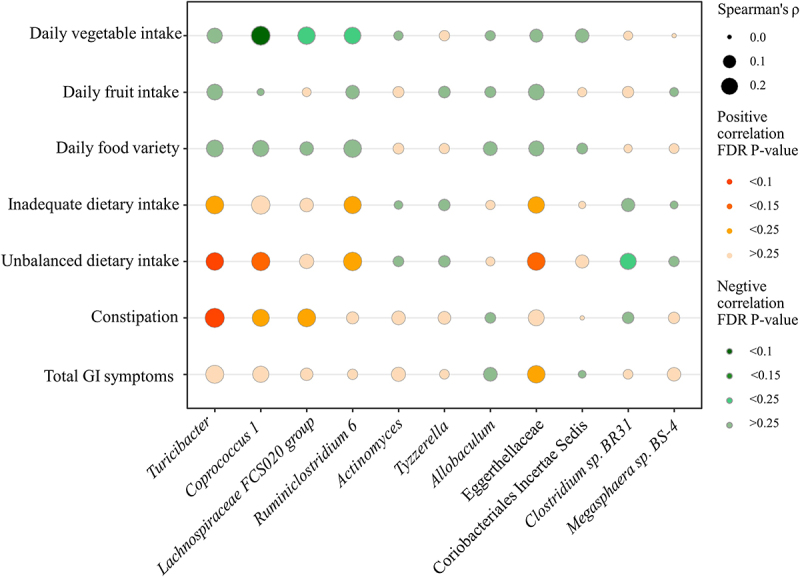
The correlation between unbalanced dietary intake and altered gut microbiota and GI symptoms in children with ASD (*n* = 90). GI symptoms include constipation and total GI symptoms. The size of the bubble represents the correlation levels. The color of the bubble represents the FDR-corrected P-value levels. Orange: a positive correlation. Green: a negative correlation. Adjusted for child’s age, sex, BMI, ASD symptom severity, intellectual functioning, birth mode, feeding patterns before 2 years of age, antibiotic exposure before the age of 5, daily sleep duration, daily MVPA duration, maternal obesity, and GI symptoms during pregnancy. GI, gastrointestinal; ASD, autism spectrum disorder; BMI, body mass index; MVPA, moderate-to-vigorous physical activity.

In this study, the dietary intake (food groups intakes, food variety, and diet quality) were compared between children with ASD and TD children. The results were consistent with or without adjustment of the covariates that children with ASD consumed fewer vegetables/fruits, less variety of food, and a higher degree of inadequate/unbalanced dietary intake than TD children (Supplementary Tables S3, 4). Among the 11 altered microbiota, the daily vegetable intake was negatively associated with *Coprococcus 1* and *Lachnospiraceae FCS020 group* (*r* = −0.297, FDR corrected P-value = 0.088; *r* = −0.238, FDR corrected P-value = 0.198) (Figure 4, Supplementary Table S7). Inadequate dietary intake was positively correlated with the *Turicibacter* and Eggerthellaceae (*r* = 0.241, FDR corrected P-value = 0.187; *r* = 0.197, FDR corrected P-value = 0.234). Unbalanced dietary intake was positively correlated with the *Turicibacter*, *Coprococcus 1*, and Eggerthellaceae (*r* = 0.234, FDR corrected P-value = 0.099; *r* = 0.257, FDR corrected P-value = 0.121; *r* = 0.240, FDR corrected P-value = 0.125) (Figure 4, Supplementary Table S7); Unbalanced dietary intake was negatively correlated with *Clostridium sp. BR31* (*r* = −0.195; FDR corrected P-value = 0.200) (Figure 4, Supplementary Table S7).

### The correlation between altered gut microbiota and GI symptoms in children with ASD

In this study, the GI symptoms (constipation and total GI symptoms scores) were also compared between children with ASD and TD children. The results were consistent with or without adjustment of the covariates that children with ASD had more severe constipation and total GI symptoms than TD children (Supplementary Tables S5, 6). Among the 11 altered microbiota, the *Turicibacter*, *Coprococcus 1*, and *Lachnospiraceae FCS020 group* were positively correlated with constipation (*r* = 0.291, FDR corrected P-value = 0.099; *r* = 0.213, FDR corrected P-value = 0.242; *r* = 0.241, FDR corrected P-value = 0.165) (Figure 4, Supplementary Table S7). The Eggerthellaceae was positively correlated with total GI symptoms (*r* = 0.228, FDR corrected P-value = 0.242) (Figure 4, Supplementary Table S7).

### The altered gut microbiota modulated the associations of unbalanced dietary intake with GI symptoms in children with ASD

**Figure 5.**
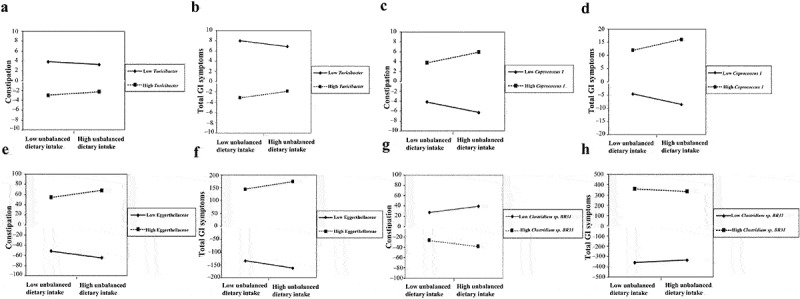
The altered gut microbiota modulated the associations of unbalanced dietary intake with GI symptoms in children with ASD (*n* = 90). GI symptoms include constipation and total GI symptoms. Adjusted for child’s age, sex, BMI, ASD symptom severity, intellectual functioning, birth mode, feeding patterns before 2 years of age, antibiotic exposure before the age of 5, daily sleep duration, daily MVPA duration, maternal obesity, and GI symptoms during pregnancy. GI, gastrointestinal; ASD, autism spectrum disorder; BMI, body mass index; MVPA, moderate-to-vigorous physical activity. A statistically significant modulation (P-value <0.05).

Among the altered gut microbiota, the modulating effect models adjusting for the covariates revealed that the increased *Turicibacter* (β = 0.327, P-value = 0.022; β = 0.593, P-value = 0.014), *Coprococcus 1* (β = 1.061, P-value = 0.029; β = 1.995, P-value = 0.036) and Eggerthellaceae (β = 6.732, P-value = 0.010; β = 14.452, P-value = 0.003) positively modulated the associations of unbalanced dietary intake with constipation and total GI symptoms (Table 2, Figure 5a-f), and the decreased *Clostridium sp. BR31* (β = −6.045, P-value = 0.041; β = −12.609, P-value = 0.033) negatively modulated their associations in children with ASD (Table 2, Figure 5g,h). Hence, the increased microbiota *Turicibacter*, *Coprococcus 1*, Eggerthellaceae, and the decreased *Clostridium sp. BR31* was focused on in the later analyses.

**Table 2.** The altered gut microbiota modulated the associations of unbalanced dietary intake with GI symptoms in children with ASD (*n* = 90).

Parameter	β	SE	95%CI	Value of t	P-value	Effect size
*Turicibacter*^ac^	0.197	0.127	−0.055 to 0.449	1.558	0.123	0.111
*Turicibacter*^ad^	0.327	0.140	0.048 to 0.605	2.338	0.022	0.366
*Turicibacter*^bc^	0.419	0.242	−0.063 to 0.901	1.730	0.087	0.110
*Turicibacter*^bd^	0.593	0.237	0.122 to 1.064	2.505	0.014	0.361
*Coprococcus 1*^ac^	0.816	0.452	−0.082 to 1.714	1.807	0.074	0.179
*Coprococcus 1*^ad^	1.061	0.478	0.109 to 2.013	2.221	0.029	0.372
*Coprococcus 1*^bc^	1.554	0.880	−0.195 to 3.302	1.766	0.081	0.138
*Coprococcus 1*^bd^	1.995	0.933	0.136 to 3.853	2.138	0.036	0.287
Eggerthellaceae^ac^	5.000	2.446	0.134 to 9.860	2.043	0.044	0.133
Eggerthellaceae^ad^	6.732	2.555	1.643 to 11.821	2.635	0.010	0.433
Eggerthellaceae^bc^	12.244	4.645	3.010 to 21.478	2.636	0.010	0.148
Eggerthellaceaea^bd^	14.452	4.617	5.253 to 23.651	3.131	0.003	0.479
*Clostridium sp. BR31*^ac^	−4.818	2.780	−10.345 to 0.709	−1.733	0.087	0.192
*Clostridium sp. BR31*^ad^	−6.045	2.910	−11.843 to −0.248	−2.077	0.041	0.391
*Clostridium sp. BR31*^bc^	−6.281	5.510	−17.234 to 4.673	−1.140	0.258	0.110
*Clostridium sp. BR31*^bd^	−12.609	5.792	−24.190 to −1.028	−2.177	0.033	0.855

GI symptoms include constipation and total GI symptoms. ^a^ Constipation was the dependent variable. ^b^ GI symptoms were the dependent variable. Constipation and total GI symptoms were evaluated by subscale and total scores of the 6-item gastrointestinal severity index (6-GSI). Unbalanced dietary intake was evaluated by diet quality distance (DQD). ^c^ Crude model. ^d^ Adjusted for child’s age, sex, BMI, ASD symptom severity, intellectual functioning, birth mode, feeding patterns before 2 years of age, antibiotic exposure before the age of 5, daily sleep duration, daily MVPA duration, maternal obesity, and GI symptoms during pregnancy. Effect size = Cohen’s f^2^ for moderating effect models. GI, gastrointestinal; ASD, autism spectrum disorder; SE, Standard error; BMI, body mass index; MVPA, moderate-to-vigorous physical activity. Statistically significant modulation (P-value <0.05).

### The correlation between altered gut microbiota and altered gut metabolites in children with ASD

**Figure 6.**
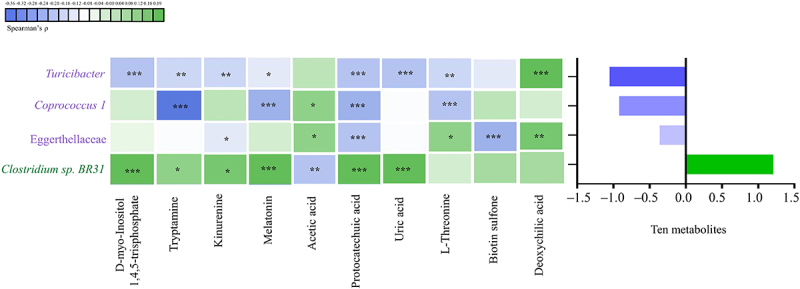
The correlation between altered gut microbiota and altered gut metabolites in children with ASD (*n* = 90). The graph on the right shows the total partial Spearman coefficients between gut microbiota and ten altered metabolites. The altered gut microbiota were the bacterial taxa modulating the associations of unbalanced dietary intake with GI symptoms. Adjusted for child’s age, sex, BMI, intellectual functioning, birth mode, feeding patterns before 2 years of age, antibiotic exposure before the age of 5, daily sleep duration, daily MVPA duration, maternal obesity, and GI symptoms during pregnancy. ASD, autism spectrum disorder; GI, gastrointestinal; BMI, body mass index; MVPA, moderate-to-vigorous physical activity. * FDR corrected P-value <0.25, ** FDR corrected P-value <0.1, *** FDR corrected P-value <0.05.

As for the correlations between altered gut microbiota and metabolites, we found that the increased *Turicibacter*, *Coprococcus 1*, and Eggerthellaceae showed an overall negative correlation with the ten altered metabolites (FDR corrected P-value <0.25). In contrast, the decreased *Clostridium sp. BR31* showed an overall positive correlation with the ten altered metabolites (FDR corrected P-value <0.25). See Figure 6 and Supplementary S8 for the specific correlations of each altered microbiota and metabolite.

### The correlation between altered gut metabolites and GI symptoms in children with ASD

**Figure 7.**
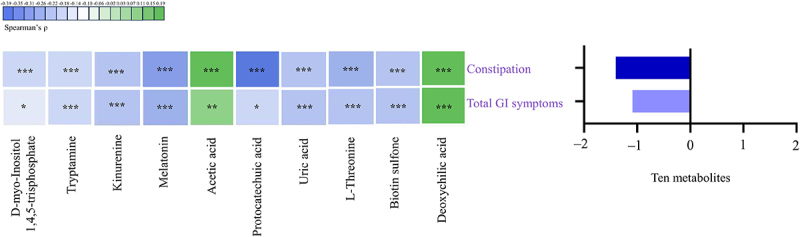
The correlation between altered gut metabolites and GI symptoms in children with ASD (*n* = 90). GI symptoms include constipation and total GI symptoms. The graph on the right shows the total partial Spearman coefficients between the ten altered metabolites and GI symptoms. Adjusted for child’s age, sex, BMI, intellectual functioning, birth mode, feeding patterns before 2 years of age, antibiotic exposure before the age of 5, daily sleep duration, daily MVPA duration, maternal obesity, and GI symptoms during pregnancy. GI, gastrointestinal; ASD, autism spectrum disorder; BMI, body mass index; MVPA, moderate-to-vigorous physical activity. * FDR corrected P-value <0.25, ** FDR corrected P-value <0.1, *** FDR corrected P-value <0.05.

**Figure 8.**
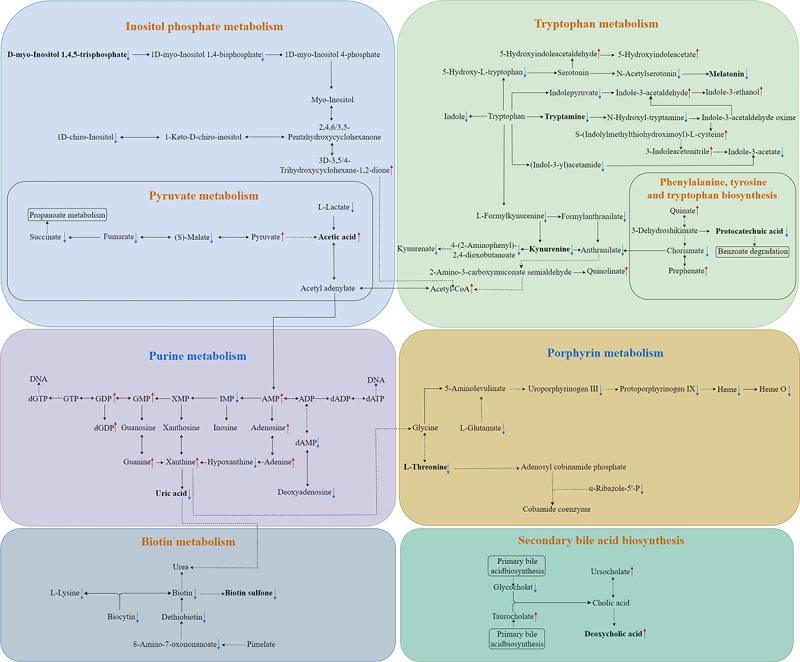
Ten altered gut metabolites correlated with GI symptoms in the eight KEGG pathways. GI symptoms include constipation and total GI symptoms. 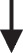 The direct pathway according to the KEGG database. 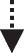 The indirect pathway according to the KEGG database. 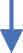 Decreased gut metabolites, fold change ≥1.1, variable importance in projection (VIP) ≥ 1.25, and P-value <0.05.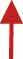 Increased gut metabolites, fold change ≤0.9, VIP ≥ 1.25, and P-value <0.05. Ten metabolites derived from microbiota (*Turicibacter*, *Coprococcus 1*, Eggerthellaceae, and *Clostridium sp. BR31*) were screened out, enriched in eight metabolic pathways, and were identified to correlate with GI symptoms in ASD children. GI, gastrointestinal; ASD, autism spectrum disorder.

**Figure 9.**
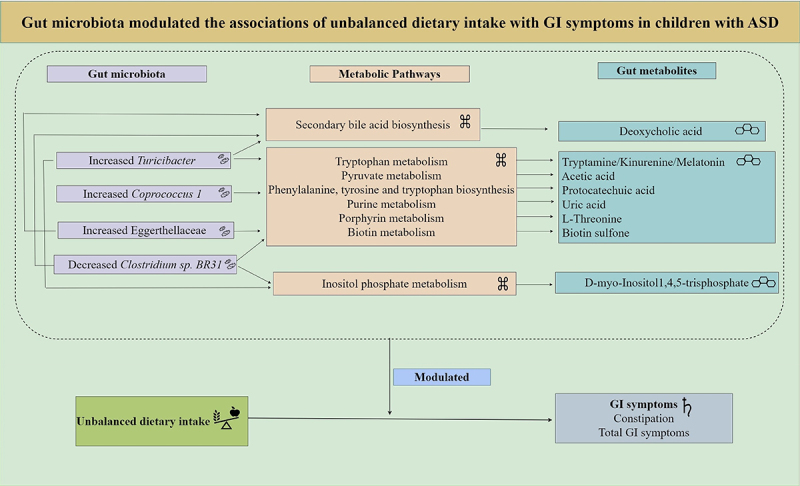
The summary of the modulating role of gut microbiota on the associations of unbalanced dietary intake with GI symptoms in children with ASD.

GI, gastrointestinal; ASD, autism spectrum disorder.

Among the altered metabolites, ten metabolites derived from microbiota (*Turicibacter*, *Coprococcus 1*, Eggerthellaceae, and *Clostridium sp. BR31*) were screened out, enriched in eight metabolic pathways, and were identified to correlate with constipation and total GI symptoms in ASD. The ten altered gut metabolites included eight decreased and two increased metabolites (Supplementary Figure S3, Supplementary Table S9). Notably, the eight decreased metabolites, namely d-myo-Inositol 1,4,5-trisphosphate, tryptamine, kynurenine, melatonin, protocatechuic acid, uric acid, L-Threonine, and biotin sulfone, showed significantly negative correlations with constipation and total GI symptoms (FDR corrected P-value <0.25) (Figure 7, Supplementary Table S10). The two increased metabolites, including acetic acid and deoxycholic acid, showed significantly positive correlations with constipation and total GI symptoms (FDR corrected P-value <0.1) (Figure 7, Supplementary Table S10). In summary, these ten altered metabolites exhibited an overall negative correlation with GI symptoms in children with ASD (Figure 7). Besides, the eight metabolic pathways including Inositol phosphate metabolism, Tryptophan metabolism, Pyruvate metabolism, Phenylalanine, tyrosine and tryptophan biosynthesis, Purine metabolism, Porphyrin metabolism, Biotin metabolism, and Secondary bile acid biosynthesis were exhibited (Figures 3d and 8). Therefore, combined microbiome and metabolome analyses revealed a modulating role of gut microbiota on the associations of unbalanced dietary intake with GI symptoms in ASD (Figure 9).

## Discussion

Our study observed that there existed 11 significantly altered gut microbiota (FDR corrected P-value <0.05) and 397 altered metabolites (P-value <0.05) in children with ASD compared with TD children. Among 11 altered gut microbiota, the increased *Turicibacter*, *Coprococcus 1*, and Eggerthellaceae positively modulated the associations of unbalanced dietary intake with constipation and total GI symptoms and the decreased *Clostridium sp. BR31* negatively modulated their associations in children with ASD (P-value <0.05). Among the altered metabolites, ten metabolites derived from microbiota (*Turicibacter*, *Coprococcus 1*, Eggerthellaceae, and *Clostridium sp. BR31*) correlated with constipation and total GI symptoms in ASD (FDR corrected P-value <0.25). Together, the altered microbiota strengthens the relationship between unbalanced dietary intake and GI symptoms in ASD. Further, the metabolomics findings support the modulating roles of gut microbiota on the associations of unbalanced dietary intake with GI symptoms in children with ASD.

Herein, we noticed 11 altered gut microbiota, including the increased abundance of *Turicibacter*, *Coprococcus 1*, *Lachnospiraceae FCS020 group*, *Allobaculum*, Eggerthellaceae, Coriobacteriales Incertae Sedis, and *Megasphaera sp. BS-4*, and the decreased abundance of *Ruminiclostridium 6*, *Actinomycetales*, *Tyzzerella*, and *Clostridium sp. BR31* in children with ASD relative to TD children (FDR corrected P-value <0.05). *Turicibacter* displayed the highest increase in abundance between children with ASD and TD children in the current study (FDR corrected P-value <0.001). Similarly, it has been reported that *Turicibacter* was associated with other neurological disorders, such as Parkinson’s disease and depression (P-value <0.05).^34,35^ *Turicibacter* as a potential pathobiont and its interaction with the host are poorly understood, and thus its basic pathogenic mechanisms need to be further investigated. *Coprococcus 1* belongs to the Lachnospiraceae family and can produce SCFAs^36^. SCFAs are the fundamental gut microbial byproducts that are related to microbiome-gut-brain axis function. SCFAs mainly include acetic acid (AA), propionic acid (PPA), butyrate (BTA), and valeric acid (VA)^37^. Growing evidence also shows that higher concentrations of SCFAs may be plausible environmental agents that are related to altered GI function and overall health status in children with ASD.^38^ Interestingly, the *Lachnospiraceae FCS020 group*, as a member of the Lachnospiraceae family, can also increase the production of SCFAs.^39^ *Allobaculum* isolates from ulcerative colitis patients exacerbate colitis in mice, implying that *Allobaculum* species may play causal roles in disease in a subset of Inflammatory Bowel Disease (IBD) patients.^40^ Sadik et al. found evidence of a potential causal link between parental, particularly maternal, IBD and autism.^41^ Therefore, the relationship between *Allobaculum* and autism deserves extensive exploration. It has been recognized that the abundance of Eggerthellaceae affected lipid metabolism in patients with radiation enteritis.^42^ The lipid metabolism status and its end-products can impact the host’s systemic inflammation.^43^ *Ruminiclostridium 6*, displaying lower abundance in the ASD group of this study (FDR corrected P-value = 0.034), was negatively correlated with the inflammation-related miR-155-5p in the proximal colon and midbrain tissues (P-value <0.05),^44^ and strong inflammation states are associated with ASD.^45^ Our analysis illustrated a significantly lower relative abundance of *Actinomyces* in ASD (FDR corrected P-value = 0.002). Nevertheless, *Actinomyces* showed discrepancies at a statistically significant level regarding its abundance in children with ASD and GI symptoms compared to their control groups.^46^ Our study also manifested a significantly lower abundance of *Tyzzerella* in ASD children (FDR corrected P-value <0.001). Similar results were reported in 2019 by our colleagues (FDR corrected P-value = 0.002).^47^ Accordingly, another study suggested that *Tyzzerella* was negatively associated with chronic intestinal inflammation in part of the intestine, showing this microbiota might play as probiotics in irritable bowel syndrome (IBS) patients. (P-value <0.05).^48^ Finegold et al. recorded nine *Clostridium* species in the stool of autistic children that were not found in fecal samples from healthy subjects.^49^ In contrast, we noted a significantly decreased *Clostridium sp. BR31* in the ASD group (FDR corrected P-value <0.001). The reason for these disparities needs to be further explored.

As for the comparisons of diets between children with ASD and TD children, our findings are in agreement with some prior works that autistic children consumed fewer vegetables/fruits than did TD children.^50–52^ In addition to lower vegetables/fruit consumption, a less variety of food and inadequate/unbalanced dietary intake were observed among ASD children in the present study (P-value <0.05). A study^53^ from Hong Kong confirmed our findings that there was less variety of food (P-value = 0.005) and poorer overall diet quality (P-value = 0.001) in children with ASD than in TD children. Sharp et al. documented that 78.5% of children with ASD consumed a diet at risk for five or more inadequacies.^52^ Dietary problems in children with ASD are of particular concern because they might cause or aggravate inadequate nutrient intakes.

Children with ASD tend to eat an unbalanced dietary intake, which can have a fundamental effect on the gut microbiota and its metabolites. In our study, the daily vegetable intake was negatively associated with *Coprococcus 1* (FDR corrected P-value = 0.088). The researchers have pointed out that vegetable intake was positively associated with the relative abundance of the *Lachnospira* genus (FDR corrected P-value = 0.032).^54^ Collectively, these studies highlight the important role of vegetable intake in the abundance of specific microbiota. Besides, we identified that unbalanced dietary intake was positively correlated with the *Turicibacter*, *Coprococcus 1*, and Eggerthellaceae, as well as negatively correlated with the *Clostridium sp. BR31* (FDR corrected P-value <0.25). Indeed, it has been documented that diet is one of the most influential environmental factors in determining the composition of the gut microbiota.^21^ In light of the extant literature and the findings of the current study, it appears to be more convincing to confirm the associations of dietary intake with the gut microbial signature. Moreover, unhealthy eating patterns-induced changes in gut microbiota composition can lead to an increased risk of developing certain diseases (e.g., IBD and CVD).^55^ Coincidentally, the present study observed that the children with ASD exhibited more severe constipation and total GI symptoms than TD children (P-value <0.05).

It is worth noting that the altered microbiota, mainly including *Turicibacter*, *Coprococcus 1*, and Eggerthellaceae, positively modulated the associations of unbalanced dietary intake and constipation and total GI symptoms in children with ASD (P-value <0.05). Lynch et al. indicated that *Turicibacter* could lead to changes in host bile acid profiles.^56^ Impaired bile acids homeostasis can affect the intestine, contributing to the pathogenesis of GI diseases.^57^ Accordingly, deoxycholic acid derived from *Turicibacter*, as a bile acid, presented a higher abundance in ASD in the current study, indicating the possible abnormal status of the related second bile acid biosynthesis pathway. *Coprococcus 1* could produce SCFAs that are potentially toxic to the mitochondria, which is known to cause GI dysfunction.^27^ Interestingly, acetic acid, the most abundant SCFAs in the human colon, has been observed to be higher in ASD in our study, which might be a possible mechanism of GI problems in ASD. As mentioned earlier, the Eggerthellaceae could affect lipid metabolism,^42^ thereby affecting the integrity of the intestinal barrier,^42^ supporting an association of Eggerthellaceae with GI symptoms. The abundance of *Clostridium* was positively correlated with the content of 5-Hydroxytryptamine (serotonin) in the colon.^58^ Serotonin, an important product of tryptophan metabolism, is involved in regulating GI motility and promoting intestinal peristalsis by binding to serotonin receptors expressed in the colon epithelial cells.^58^ Interestingly, we observed a lower abundance of *Clostridium sp. BR31* in ASD children (FDR corrected P-value <0.001). And we noticed a lower abundance of vital metabolites in the tryptophan metabolic pathway, including tryptamine, kynurenine, and indole, although no decreases in tryptophan and serotonin were directly observed. These findings may mechanistically explain, in part, the negative modulation of decreased *Clostridium sp. BR31* on the associations of unbalanced dietary intake with GI symptoms in ASD.

The gut microbiota may influence various processes of GI pathophysiology in ASD via the key metabolic molecules.^9,20,29^ We identified ten altered metabolites (including d-myo-Inositol 1,4,5-trisphosphate, tryptamine, kinurenine, melatonin, protocatechuic acid, uric acid, L-Threonine, biotin sulfone, acetic acid, and deoxycholic acid) derived from *Turicibacter*, *Coprococcus 1*, Eggerthellaceae, and *Clostridium sp. BR31* correlating with GI symptoms (FDR corrected P-value <0.25). D-myo-Inositol 1,4,5-trisphosphate, as a second messenger, can bind to its own intracellular receptor causing the release of Ca^2+,59^ which can contribute to the pathogenesis of various diseases.^60^ On this basis, we infer that constipation in ASD may be a result of aberrant gut motility caused by decreased D-myo-Inositol 1,4,5-trisphosphate-mediated calcium imbalance. Tryptophan can be metabolized into tryptamine, kynurenine, and indole, thereby modulating neuroendocrine and intestinal immune responses.^61^ A previous study implicated that patients with ASD exhibited reduced levels of tryptophan in the plasma.^62^ One study validated that tryptamine can increase the fluid secretions in colonoids from germ-free and humanized mice, as well as improve gut motility in germ-free mice.^63^ Kynurenate may have anti-inflammatory properties in the GI tract^64^ and can inhibit the proliferation of colon cancer cells in vitro.^65^ Indoles can contribute to maintaining the intestinal biological barrier, affecting the immune system’s function, and markedly improving intestinal health.^66^ Melatonin is another decreased metabolite present in the tryptophan metabolism for ASD children, achieving antioxidant and anti-inflammatory functions locally and regulating gut motility, and therefore the reduction of melatonin may be one of the vital drivers for GI problems in ASD.^67^ Thus, the metabolites including tryptamine, kynurenine, and melatonin exhibited negative correlations with GI symptoms in this study (FDR corrected P-value <0.1). Protocatechuic acid had a protective effect on oxidative stress, inflammation, and intestinal barrier function by modulating intestinal flora.^68^ Uric acid, as a powerful antioxidant, plays a defensive role in intestinal injury via oxidative stress elimination and microbiota composition modulation, preferably in gut immunity.^69^ L-Threonine and attapulgite can maintain gut health by increasing the levels of intestinal barrier protein expression and regulating the balance of gut microbiota.^70^ Biotin plays an extremely important role in maintaining intestinal homeostasis and microbial composition balance.^71^ Therefore, lack of protection caused by the reduction of beneficial metabolites like protocatechuic acid, uric acid, L-Threonine, and biotin may be one of the underlying causes of GI issues for ASD children. Additionally, we noticed increased acetic acid and deoxycholic acid positively correlating with GI symptoms (FDR corrected P-value <0.1). It was speculated that the increased intestinal permeability presented in ASD could be related to changes in the fecal production of acetic acid.^38^ It was described that deoxycholic acid can induce apoptosis of gastric epithelial cell line GES-1^72^ and can potentiate the mechanosensitivity of high threshold spinal afferents leading to visceral hypersensitivity.^73^

The present study was integrated into a comprehensive assessment project, which may reduce parents’ recruitment bias because of their interest in their child’s dietary and GI conditions. All subjects in this study were recruited based on research projects, not outpatients or inpatients, which may minimize the admission rate bias. To diminish the investigation bias, all investigators were trained strictly, and the questionnaires filled out by parents were checked and reviewed by the trained investigators. There are also some limitations of our study. First, the ASD children who had neuropsychiatric disorders or received special diets set by parents were excluded from this study, and future studies will capture the ASD children with co-occurring neuropsychiatric conditions and/or receiving dietary interventions for intense investigation. Second, we are limited by taxonomic classification based on 16S rRNA profiling rather than shotgun metagenomic sequencing, as 16S rRNA gene sequencing only captured a portion of the gut microbiota community identified by shotgun sequencing.^74^ Third, relying on parents’ reports on FFQ can be subject to social desirability bias and recall bias,^75^ and future studies are encouraged to apply reproducible metabolomics biomarkers as surrogates to validate parent-reported diet measures.^76^ Fourth, we just performed the correlation analyses to study the metabolites correlating with GI symptoms, and future studies will more deeply explore the mechanisms of gut metabolites along the metabolism pathway on GI symptoms in ASD through molecular biology and animal experiments.

In conclusion, results from the present study demonstrated that the altered microbiota strengthens the relationship between unbalanced dietary intake and GI symptoms in ASD. Furthermore, the metabolomics findings support the modulating role of gut microbiota on the associations of unbalanced dietary intake with GI symptoms in children with ASD. The present findings suggest that not only the dietary intervention but also the gut microbiota- and metabolites-targeted interventions should be designed to alleviate GI symptoms in children with ASD. Nevertheless, more research is warranted to replicate our findings and to further investigate the modulating role of gut microbiota on the associations of diet with GI symptoms in ASD.

## Materials and methods

### Study design and participants

An age- and sex-matched (1:1) case-control study was carried out in China between September 2021 and May 2022. The children with ASD and TD children from 2 to 10 years were recruited based on the strict eligibility and exclusion criteria from Guangzhou and Foshan City, Guangdong Province, China (Figure 1). The diagnosis of ASD was confirmed with the Autism Diagnosis Observation Schedule-2 (ADOS-2)^77^ according to the Diagnostic and Statistical Manual of Mental Disorders, 5th Edition (DSM-5)^78^ criteria by two experienced developmental and behavioral pediatricians. The Social Communication Questionnaire (SCQ)^79^ was used to screen for a potential diagnosis of ASD for TD children, and the scores were within the typical range (Supplementary Table S11). Also, none of the TD children has a sibling with a confirmed diagnosis of ASD. Besides, the exclusion criteria for ASD and TD children were: had a prior diagnosis of neuropsychiatric disorders [e.g., seizures, anxiety disorder, depressive disorder, attention deficit hyperactivity disorder (ADHD), schizophrenia, or bipolar disorder] and clinically evident inflammatory conditions (e.g., Crohn’s disease and ulcerative colitis); received a special diet (including the gluten-free/casein-free diet and the ketogenic diet) set by parents, took probiotics, prebiotics, or synbiotics supplements, antibiotics or antifungal medications, and laxatives within three months prior to stool sample collection.

### Sample size

A matched case-control design was adopted in this study. The case group was ASD children and the control group was TD children. The matching principle was that the age difference between the case and control was less than 1.5 years. It was reported that GI symptoms have been found to occur in 10% of TD children.^80^ Children with ASD are 2.5 times more likely to suffer from GI symptoms than TD children.^80^ The correlation coefficient between cases and their matched controls (phi) was 0.2.^81^ At least 76 ASD cases and 76 controls were calculated by sample size calculation software to provide 90% statistical power (1-β) and a 2-tailed 5% α level. After considering the 85% qualified rate of questionnaire filling, this study finally enrolled 90 ASD cases and 90 TD controls with matched age and sex.

### Dietary and GI assessment

Dietary data were obtained from parents of children by using a validated 7-day food frequency questionnaire (FFQ).^82^ The FFQ consisted of food lists [cereals/potatoes, vegetables, fruit, livestock/poultry meat, fish/shrimp, eggs, soybeans, dairy, and drinking water]. The FFQ with the number of times during the past seven days for each food and the average quantity of food consumed each time based on a specified standardized portion size (a serving = 50 grams) was used to calculate the average daily food intake (g/ml per day). The diet quality was assessed using the Dietary Balance Index (DBI) established based on the recommended daily food intake from the Dietary Guidelines for Chinese Residents (2022) and Chinese Food Pagoda. The specific methods for diet quality assessment via DBI had been described in the previously published study.^83^ The DBI consists of three main evaluation indexes, namely high bound score (HBS), low bound score (LBS), and diet quality distance (DQD). HBS is the sum of all positive values, with higher scores reflecting the degree of excessive dietary intake. LBS is the sum of absolute values of negative scores, with higher scores indicating the degree of insufficient dietary intake. DQD is the sum of HBS and LBS, with higher scores representing the degree of unbalanced dietary intake. The severity of GI symptoms was assessed using a shortened version of the 6-item Gastrointestinal Severity Index (6-GSI) questionnaire,^84^ composed of six symptoms, including constipation, diarrhea, stool consistency, stool smell, flatulence, and abdominal pain. Each symptom is rated on a three-point Likert-type scale ranging from 0 to 2, with a higher score signifying more severe GI symptoms.^85^ The intraclass correlation coefficient (ICC) for evaluating interrater reliability of the GI total scores was found to be high at 0.95 [95% confidence interval(CI): 0.87–0.98].^85^

#### Procedures

The demographic data were collected via validated scales/questionnaires and in-person interviews with parents of children enrolled in this study. Furthermore, face-to-face physical, behavioral, and psychological measures were carried out on the enrolled children. Informed consent was obtained for participation, and the study was conducted in accordance with the principles of the Declaration of Helsinki and the Ethical Committee of the School of Public Health, Sun Yat-Sen University (No. 067 [2022]).

#### Stool sample collection

The parents were given a stool sample collection kit and provided detailed instructions for stool sample collection. After collection at home or the research center, the stool samples with ice bags were transported by The City Delivery to the research laboratory and stored in a − 80°C freezer within 4 h for subsequent 16S rRNA gene amplicon sequencing and UPLC-MS.

### 16S rRNA gene amplicon sequencing

Genomic DNA was extracted from the stool samples using the QIAamp DNA Stool Mini Kit (QIAGEN, Germany). The concentration and purity of DNA were detected by a Nanodrop spectrophotometer (Nanodrop Technologies Inc., Wilmington, DE, USA). The V3-V4 regions of the bacterial 16S rRNA gene was amplified with primers (338F: 5′-ACTCCTACGGGAGGCAGCA-3′; 806 R: 5′-GGACTACHVGGGTWTCTAAT-3′). The sequencing library was generated using the NEBNext® Ultra™ DNA Library Prep Kit for Illumina (New England Biolabs, Ipswich, MA, USA) following the manufacturer’s protocols. The sequencing steps were conducted on the Illumina HiSeq 2500 platform (MAGIGENE Co., Ltd., Guangzhou, Guangdong Province, China). The generated paired-end reads were merged using USEARCH (version 10.0.240)^86^ and quality-filtered by fastp (version 0.14.1).^87^ Sequences were clustered at a 97% similarity identity level by UPARSE (V11)^88^ to generate operational taxonomic units (OTUs) and were subjected to singletons removal and rarefaction before subsequent analysis. Finally, a representative sequence for each OTU was selected and assigned using the RDP Bayes-Classifier^89^ with a confidence cutoff of 80%.

The sequencing depths were examined by plotting the rarefaction curve of richness using Usearch-alpha_div_rare (V10, http://www.drive5.com/usearch/). For alpha diversity, the microbial richness and diversity were evaluated by the Chao1, Shannon_2, and Simpson indexes at phylum and genus levels with the Wilcoxon rank-sum tests. The Chao1, Shannon_2, and Simpson indexes were calculated using Usearch-alpha_div (V10, http://www.drive5.com/usearch/). For microbiota beta diversity, PCoA and NMDS based on Bray-Curtis distance, PCoA based on unweighted Unifrac and weighted Unifrac distances with PERMANOVA tests were used to evaluate the spatiotemporal variations in species composition by using the vegan package (https://www.researchgate.net/publication/258996451) in R software.^90^ The top 375 microbial OTUs were selected for microbial network analysis based on the Spearman correlation algorithms to explore co-occurrence and interaction patterns in microbial communities. Each node presents one OTU, and the node size indicates the relative abundance of each OTU. The straight line represents a significant correlation (−0.4 > *r*  > 0.4) between two nodes, and the network visualization was conducted by the “Gephi” interactive platform.^91^ Next, the significantly differential relative abundance of microbiota at family, genus, and species levels between children with ASD and TD children was tested by Wilcoxon rank-sum and ANCOM-BC tests^92^. The P-values were adjusted by Benjamini – Hochberg (BH) correction. Finally, the LEfSe and random forest model analyses were used for statistical analysis to identify the bacterial taxa differentially represented between children with ASD and TD children, and the LEfSe and random forest analyses were performed using the LEfSe software with LDA > 3.0^93^ and randomForest package in R software (version 3.6.1), respectively.

#### Untargeted metabolomic analysis using UPLC-MS

The stool samples were detected via UPLC-MS (Agilent Technologies, Santa Clara, CA, USA) using a Waters Acquity UPLC HSS T3 C18 column (2.1 mm × 100 mm, 1.8 μm) at a flow rate of 0.4 mL/min and a column temperature of 40°C. The mobile phase A was H_2_O containing 0.1% formic acid and mobile phase B was acetonitrile containing 0.1% formic acid. Samples were analyzed in both positive and negative modes in an automatic pattern. The scanning range of the time-of-flight mass spectrometry (TOF MS) was 100–1,500 m/z. The Electrospray ionization (ESI) source conditions were set as follows: Ion Source Gas1 (Gas1) as 50, Ion Source Gas2 (Gas2) as 60, curtain gas (CUR) as 35, source temperature: 550°C, IonSpray Voltage Floating (ISVF) +5500/−4500 V. The original data acquired by LC-MS was converted into mzML format by ProteoWizard software. Peak extraction, peak alignment, and retention time correction were respectively performed by the XCMS program. The “SVR” method was used to correct the peak area. The peaks with a detection rate lower than 50% in each group of samples were discarded. After that, metabolic identification information was obtained by searching the laboratory’s self-built database, integrated public database, artificial intelligence database, and MetDNA.

Volcano plots were used to summarize the results of significantly differential metabolites with VIP ≥ 1 and P-value <0.05. Two vertical lines of the volcano plots indicate metabolites log2foldchange −1 and 1, respectively, and the horizontal line indicates the P-value of 0.05. The color of the dot represents the P-value levels and the volcano plots were depicted with ggplot2 in R software (version 4.1.0). The log2-transformed relative abundance of the top 40 significantly altered gut metabolites with fold change ≤ 0.5 or ≥ 2, VIP ≥ 1, and P-value <0.05 between children with ASD and TD children were presented by the heatmap. The MPEA was carried out to find the key KEGG pathways with the significantly altered metabolites (fold change ≥ 1.1 or ≤ 0.9, VIP ≥ 1.25, and P-value <0.05) through R package clusterProfiler (version 3.8.1). Notably, the metabolites derived from microbiota were annotated using the KEGG Compound database and mapped to the KEGG Pathway database (http://www.kegg.jp/kegg).

### Statistical analyses

The normality of data was evaluated with the Kolmogorov-Smirnov test. Continuous variables were expressed as median (IQR) and analyzed by the Mann-Whitney U test. Categorical variables were expressed as percentages and analyzed by the chi-squared test. Variables in dietary intake and GI symptoms were compared between children with ASD and TD children by applying the linear regression models adjusting for the covariates (see Supplementary Table S1 for details of the covariates). The Spearman partial correlation coefficients with the Spearman’s P-value and BH correction were used to gain insight into the correlation between unbalanced dietary intake and altered gut microbiota and GI symptoms, as well as the correlation between altered gut microbiota and altered gut metabolites and GI symptoms in ASD. Following, we performed the modulating effects analyses to evaluate whether altered gut microbiota could modulate the associations of unbalanced dietary intake with GI symptoms in children with ASD. Notably, collinearity diagnosis was conducted to test the potential multicollinearity among the covariates included in the models (including linear regression, Spearman partial correlation, and modulating effect models). To estimate effect sizes, the formula r=Z/√(N) was used for Mann – Whitney U-tests, effect size φ for 2 × 2 tables, and Cramer-V for tables larger than 2 × 2, with threshold values of 0.1, 0.3, and 0.5 used to categorize effects as small, medium, and large, respectively.^94^ Cohen’s f^2^ was calculated for linear regression and modulating effect models, and f^2^ ≥0.02, f^2^ ≥0.15, and f^2^ ≥0.35 were interpreted as small, medium, and large effect sizes, respectively.^95^ All data were analyzed using SPSS 26.0 (IBM Corp. Released 2019. IBM SPSS Statistics for Windows, Version 26.0. Armonk, NY, USA: IBM Corp) and R software (version 4.1.0, The R Foundation) with 2-tailed tests and P-value <0.05 was considered significant.

## Supplementary Material

Supplemental Material

## Data Availability

The datasets analyzed during the current study are available from the corresponding author upon reasonable request.
