# Supplementary figures and images for: Multi-omics analyses demonstrate the modulating role of gut microbiota on the associations of unbalanced dietary intake with gastrointestinal symptoms in children with autism spectrum disorder
Source: Gut Microbes. 2023 Nov 27;15(2):2281350. doi: 10.1080/19490976.2023.2281350 (PMC10730204; doi:10.1080/19490976.2023.2281350)

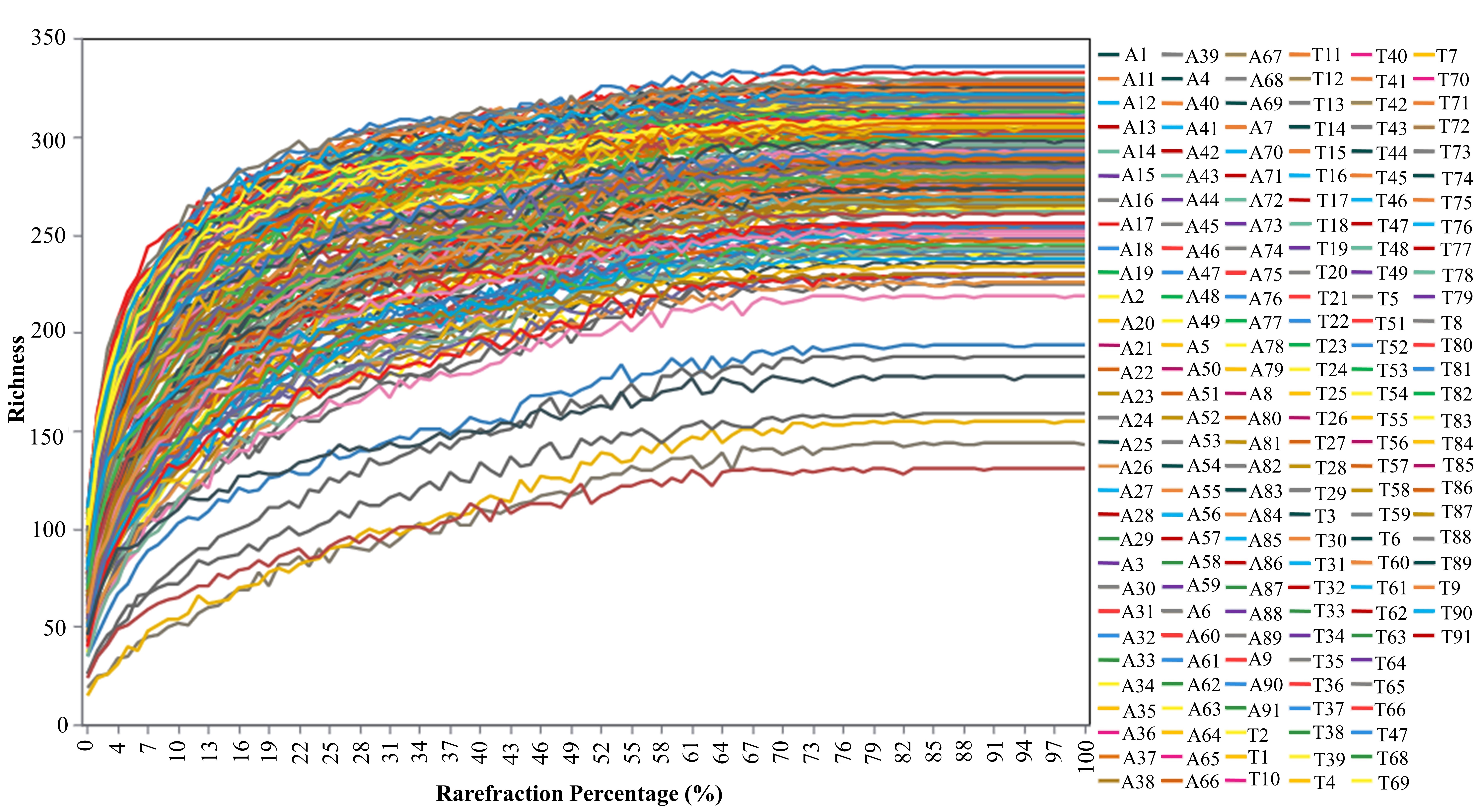

Supplement: Supplemental Material [file KGMI_A_2281350_SM1114.zip › Supplementary Figure 1 first revision.jpg]

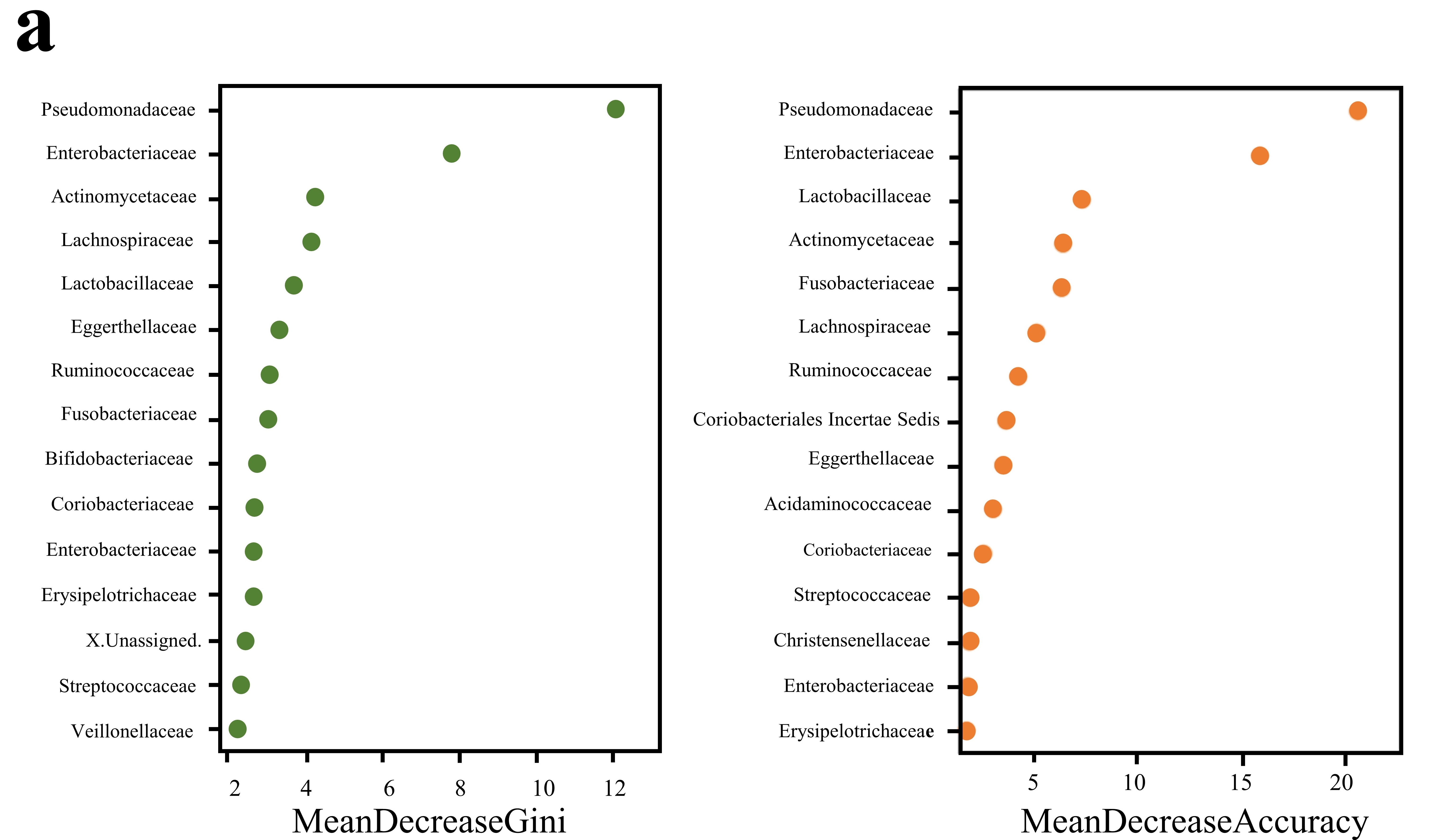

Supplement: Supplemental Material [file KGMI_A_2281350_SM1114.zip › Supplementary Figure 2a first revision.jpg]

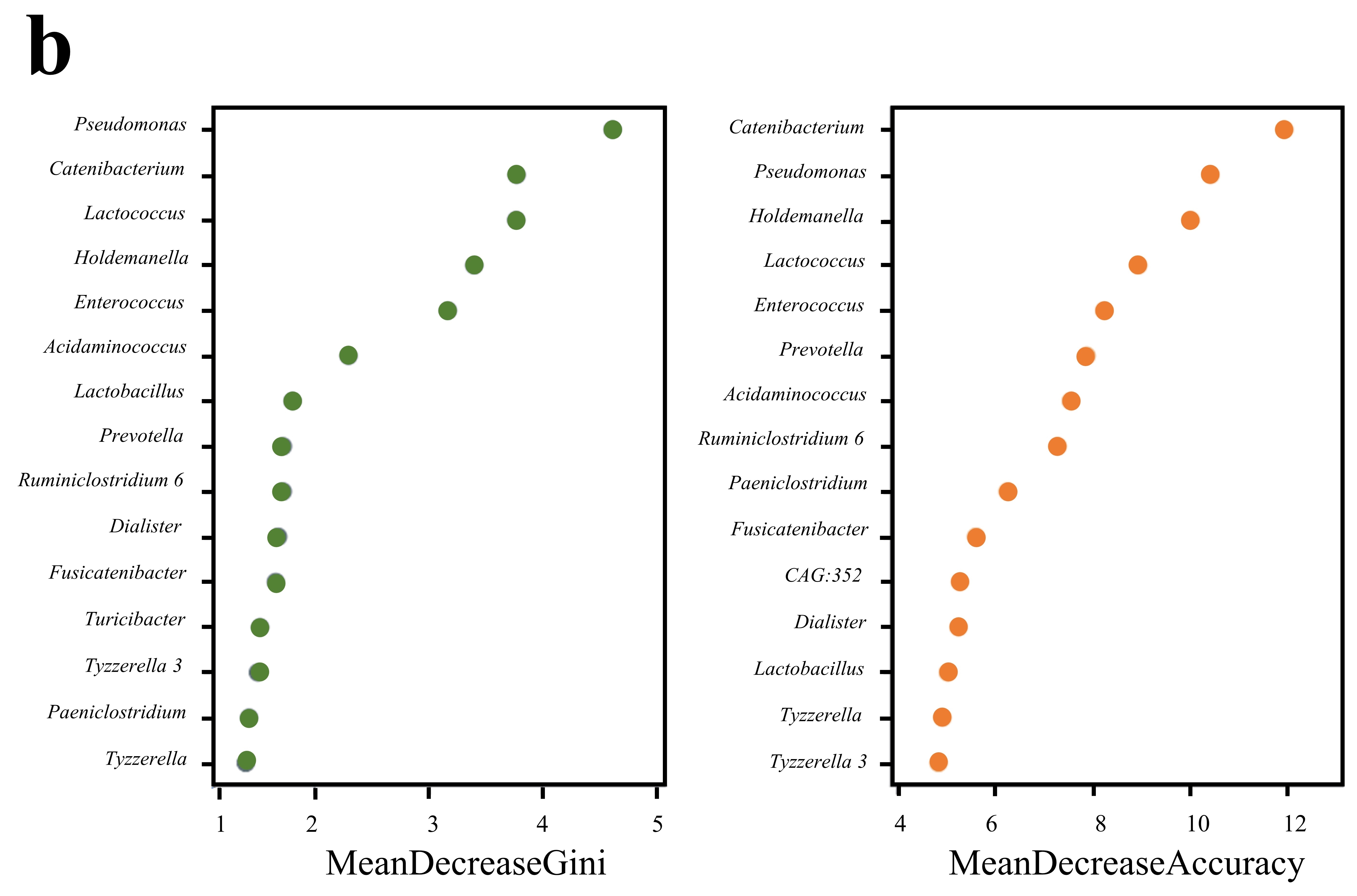

Supplement: Supplemental Material [file KGMI_A_2281350_SM1114.zip › Supplementary Figure 2b first revision.jpg]

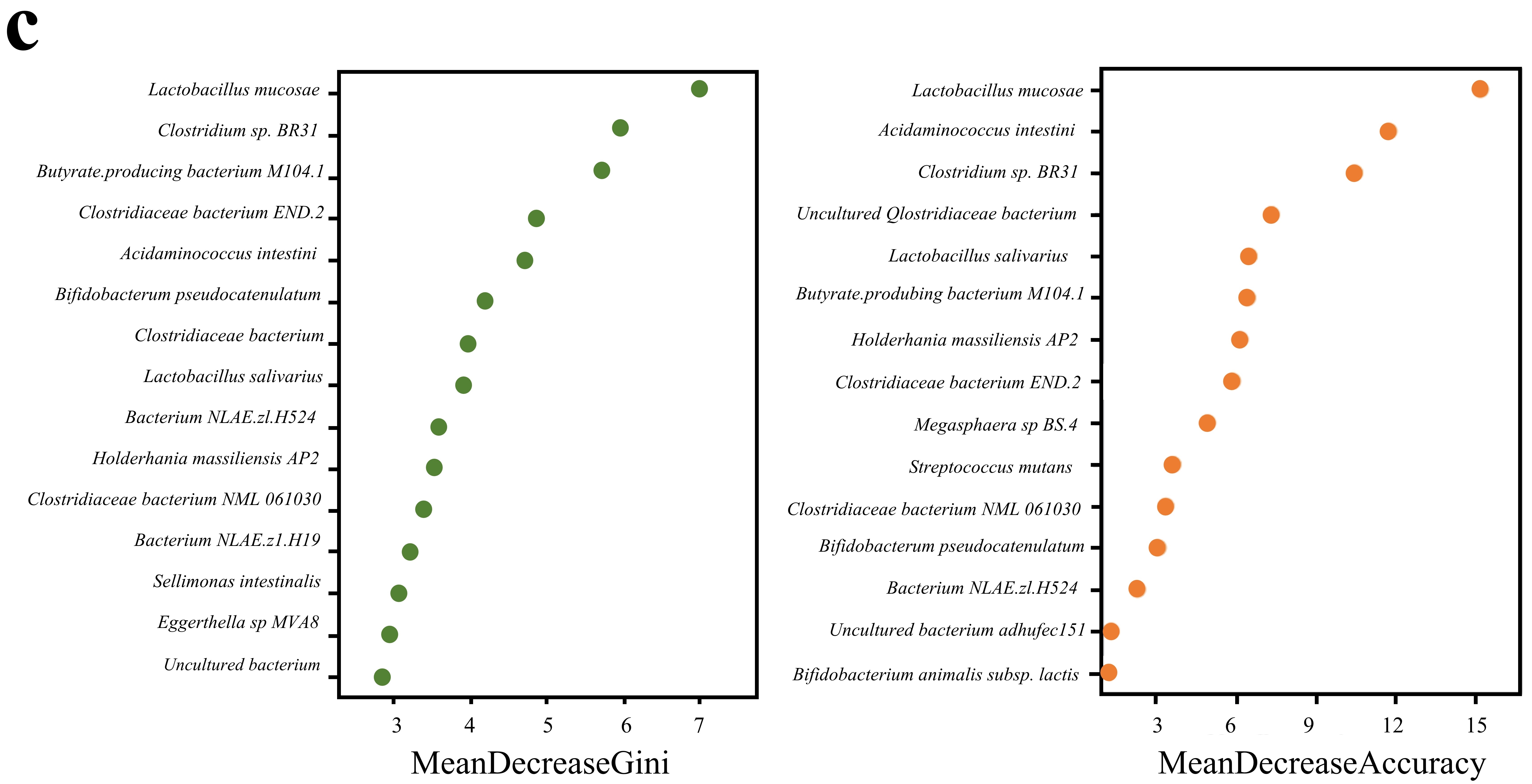

Supplement: Supplemental Material [file KGMI_A_2281350_SM1114.zip › Supplementary Figure 2c first revision.jpg]

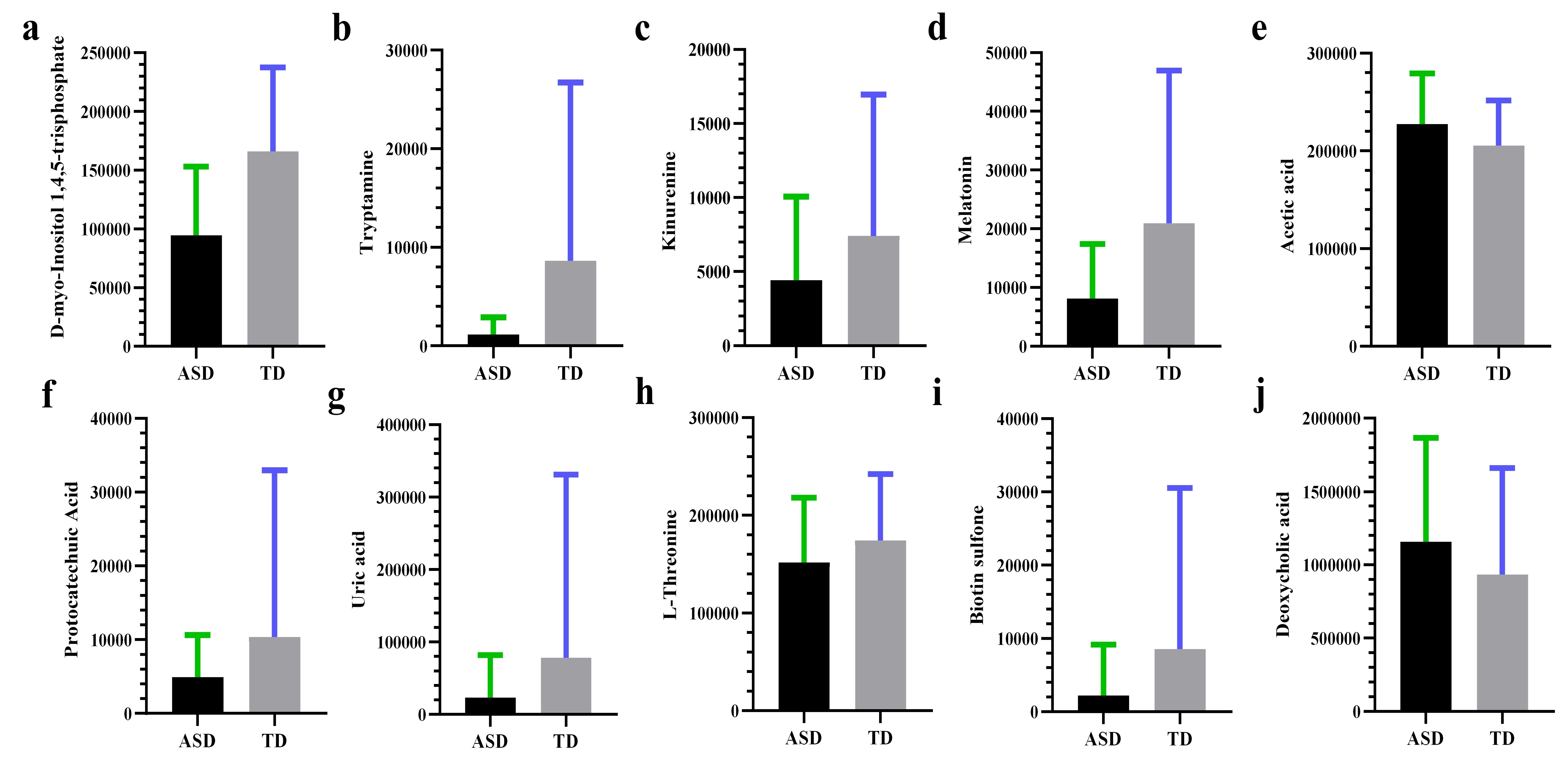

Supplement: Supplemental Material [file KGMI_A_2281350_SM1114.zip › Supplementary Figure 3 first revision.jpg]
