# Supplementary material for: Multi-omics analyses demonstrate the modulating role of gut microbiota on the associations of unbalanced dietary intake with gastrointestinal symptoms in children with autism spectrum disorder
Source: Gut Microbes. 2023 Nov 27;15(2):2281350. doi: 10.1080/19490976.2023.2281350 (PMC10730204; doi:10.1080/19490976.2023.2281350)
Supplement: Supplemental Material [file KGMI_A_2281350_SM1114.zip › Supplementary Materials first revision.doc]

**Figure S1.** **The rarefaction curve evaluating the richness and sequencing depth of gut microbiota communities in children with ASD and TD children.**

ASD, autism spectrum disorder; TD, typically developing.

**Figure S2.** **Random forest analyses of differences** **in gut microbiota between children with ASD and TD children at Family (a), Genus (b), and Species (c) levels.**

ASD, autism spectrum disorder; TD, typically developing.

**Table S1. Covariates definitions.**

| **Variables** | **Definition** | **Source** | **Type** | **Analysis format** |
| --- | --- | --- | --- | --- |
| Age | Years | Demographic questionnaire | Continuous |  |
| Sex | Sex | Demographic questionnaire | Dichotomous | Male  Female |
| Body Mass Index (BMI) | kg/m^2^ | Weight (kg)/height^2^ (m) | Continuous |  |
| ASD symptom severity | Score | Childhood Autism Rating Scale (CARS)^1^ | Dichotomous | Mild-to-moderate (score < 37)  Severe (score ≥ 37) |
| Intellectual functioning | Score | Wechsler Intelligence Scale for Children Forth Edition (WISC-IV): applied to children aged 6 to 10 years^2^  Gesell developmental Schedules (GDS): applied to children aged 2 to 5 years^3^ | Categorical | WISC-IV:  Normal (score ≥ 80)  Borderline (80> score ≥70)  Abnormal (score < 70)  GDS:  Normal (score > 85)  Borderline (85 ≥ score ≥ 76)  Abnormal (score < 76) |
| Birth mode | Birth mode | Demographic questionnaire | Categorical | Vaginal delivery  Elective caeserean section  Emergency caeserean section |
| Birth order | Birth order | Demographic questionnaire | Dichotomous | 1  >1 |
| Feeding patterns before 2 years of age | Feeding patterns | Demographic questionnaire | Categorical | Breastfeeding  Mixed feeding  Artificial feeding |
| Antibiotic exposure before the age of 5 | Antibiotic exposure | Demographic questionnaire | Dichotomous | Yes  No |
| Average daily sleep duration | Hours/day | Demographic questionnaire:  Sleep duration during the night and day | Continuous |  |
| Average daily moderate-to-vigorous physical activity time | Hours/day | Assessment questionnaire:  International Physical Activity Questionnaire Short-Form (IPAQ-SF)^4,5^ | Continuous |  |
| Maternal educational attainment | Educational attainment | Demographic questionnaire | Dichotomous | College degree or below  Undergraduate degree or higher |
| Gestational diabetes mellitus | Doctor-diagnosed | 75g oral glucose tolerance test (OGTT) administered at 24 to 28 weeks of gestation^6^ | Dichotomous | Yes  No |
| Maternal obesity | Doctor-diagnosed | [Body mass index (BMI) ≥ 30kg/m^2^]^7,8^ | Dichotomous | Yes  No |
| Gastrointestinal symptoms during pregnancy | Doctor-diagnosed | Diagnosed by the obstetrician or gastroenterologist^9^ | Dichotomous | Yes  No |
| Monthly per-capita income | Renminbi | Demographic questionnaire | Dichotomous | ≤ 8,000  > 8,000 |
| Parenting behavior | Score | Assessment questionnaire:  Parent Behavior Inventory (PBI)^10^ | Dichotomous | Support/engagement  Opposition/defiance |

**References**

1.	Kaat A, Lecavalier L. Childhood Autism Rating Scale. Encyclopedia of autism spectrum disorders. 2013;590–593.

2.	Kaufman AS, Flanagan DP, Alfonso VC, Mascolo JT. Review of Wechsler Intelligence Scale for Children, Fourth Edition (WISC-IV). Journal of Psychoeducational Assessment. 2006;24(3):278–295.

3.	Dror R, Malinger G, Ben-Sira L, Lev D, Pick CG, Lerman-Sagie T. Developmental outcome of children with enlargement of the cisterna magna identified in utero. J Child Neurol. 2009;24(12):1486–1492.

4.	Macfarlane DJ, Lee CCY, Ho EYK, Chan KL, Chan DTS. Reliability and validity of the Chinese version of IPAQ (short, last 7 days). J Sci Med Sport. 2007;10(1):45–51.

5.	Craig CL, Marshall AL, Sjöström M, Bauman AE, Booth ML, Ainsworth BE, Pratt M, Ekelund U, Yngve A, Sallis JF, et al. International physical activity questionnaire: 12-country reliability and validity. Med Sci Sports Exerc. 2003;35(8):1381–1395.

6.	International Association of Diabetes and Pregnancy Study Groups Consensus Panel, Metzger BE, Gabbe SG, Persson B, Buchanan TA, Catalano PA, Damm P, Dyer AR, Leiva Ad, Hod M, et al. International association of diabetes and pregnancy study groups recommendations on the diagnosis and classification of hyperglycemia in pregnancy. Diabetes care. 2010;33(3):676–682.

7.	Xian DM, Xu XM, Zhong JX, Liu N. Summary of best evidences on obesity management in pregnant women. Journal of Nursing Science. 2021;36(16):88–91.

8.	Tong LL, Kalish BT. The impact of maternal obesity on childhood neurodevelopment. J Perinatol. 2021;41(5):928–939.

9.	Gomes CF, Sousa M, Lourenço I, Martins D, Torres J. Gastrointestinal diseases during pregnancy: what does the gastroenterologist need to know? Ann Gastroenterol. 2018;31(4):385–394.

10.	Lovejoy MC, Weis R, O'Hare E, Rubin EC. Development and initial validation of the Parent Behavior Inventory. Psychological assessment. 1999;11(4):534–545.

| **OTU** | **Genus/Family/Species** | **Relative abundance, mean (SD)** | | | | **Wilcoxon rank-sum test** | **FDR corrected**  **P-value** | **ANCOM-BC**  **Tests** | **FDR corrected P-value^a^** |
| --- | --- | --- | --- | --- | --- | --- | --- | --- | --- |
|  |  | **ASD** | | **TD** | |  |  |  |  |
|  |  | **Mean (%)** | **SD** | **Mean (%)** | **SD** |  |  |  |  |
| OTU_35 | *Turicibacter* | 0.5855 | 0.0310 | 0.0732 | 0.0017 | –0.0051 | 0.0009 | 0.0323 | 0.0320 |
| OTU_69 | *Coprococcus 1* | 0.0925 | 0.0019 | 0.0611 | 0.0015 | –0.0003 | 0.0277 | 0.0197 | 0.0196 |
| OTU_88 | *Lachnospiraceae FCS020 group* | 0.0772 | 0.0015 | 0.0500 | 0.0011 | –0.0003 | 0.0201 | 0.1028 | 0.1648 |
| OTU_63 | *Ruminiclostridium 6* | 0.0237 | 0.0015 | 0.0868 | 0.0078 | 0.0006 | 0.0342 | 0.0097 | 0.0097 |
| OTU_115 | *Actinomyces* | 0.0218 | 0.0003 | 0.0694 | 0.0055 | 0.0005 | 0.0017 | 0.1882 | 0.1797 |
| OTU_117 | *Tyzzerella* | 0.0112 | 0.0003 | 0.0483 | 0.0016 | 0.0004 | 0.0001 | 0.1787 | 0.1111 |
| OTU_169 | *Allobaculum* | 0.0223 | 0.0016 | 1.41E-05 | 0.000001 | –0.0002 | 0.0017 | 0.0257 | 0.0255 |
| OTU_134 | Eggerthellaceae | 0.0518 | 0.0018 | 0.0517 | 0.0006 | 1.41E-06 | 0.0308 | 0.2292 | 0.2166 |
| OTU_130 | Coriobacteriales Incertae Sedis | 0.0189 | 0.0008 | 0.0065 | 0.0003 | –0.0001 | 0.0152 | 0.0420 | 0.0416 |
| OTU_105 | *Clostridium sp. BR31* | 0.0113 | 0.0007 | 0.0502 | 0.0026 | 0.0004 | 0.0003 | 0.2564 | 0.2406 |
| OTU_164 | *Megasphaera sp. BS-4* | 0.0289 | 0.0026 | 0.0022 | 0.0002 | –0.0003 | 0.0010 | 0.0519 | 0.0512 |

**Table S2. The relative abundance of altered gut microbiota between children with ASD and TD children (n = 180).**

^a^ Adjusted for child's age, sex, BMI, intellectual functioning, birth mode, feeding patterns before 2 years of age, antibiotic exposure before the age of 5, daily sleep duration, daily MVPA duration, maternal obesity, and GI symptoms during pregnancy. ASD, autism spectrum disorder; TD, typically developing; SD, standard deviation; ANCOM-BC, Analysis of Compositions of Microbiomes with Bias Correction; BMI, body mass index; MVPA, moderate-to-vigorous physical activity; GI, gastrointestinal.

**Table S3. Comparison o****f dietary intake between children with ASD and TD children (n = 180).**

| **Dietary intake** | **ASD (n = 90)** | **TD (n = 90)** | **P-value** | | **Effect Size** | |
| --- | --- | --- | --- | --- | --- | --- |
| Cereals/potatoes^a^, median (IQR) | 200 (150, 300) | 200 (150, 300) | 0.338 | 0.071 | |  |
| Vegetables^a^, median (IQR) | 100 (35.7, 155.4) | 100 (71.4, 200) | 0.025 | 0.167 | |  |
| Fruit^a^, median (IQR) | 50 (28.6, 100) | 64.3 (42.9, 100) | 0.085 | 0.128 | |  |
| Livestock/poultry meat^a^,  median (IQR) | 100 (50, 178.6) | 100 (55.4, 200) | 0.101 | 0.122 | |  |
| Fish/shrimp^a^, median (IQR) | 14.3 (6.4, 28.6) | 21.4 (9.8, 42.9) | 0.050 | 0.146 | |  |
| Eggs^a^, median (IQR) | 35.7 (14.3, 50) | 32.1 (21.4, 50) | 0.693 | 0.029 | |  |
| Soybeans^a^, median (IQR) | 7.1 (0, 15.2) | 7.1 (0, 21.4) | 0.329 | 0.073 | |  |
| Dairy^b^, median (IQR) | 250 (112.5, 402.9) | 250 (200, 400) | 0.127 | 0.114 | |  |
| Food variety^c^, mean ± SD | –5 (–6, –3) | –4 (–5, –2) | 0.004 | 0.212 | |  |
| Drinking water^b^, median (IQR) | 750 (500, 1031) | 750 (500, 1000) | 0.152 | 0.107 | |  |
| HBS, median (IQR) | 8 (4, 14) | 6 (4, 11.3) | 0.148 | 0.108 | |  |
| LBS, mean ± SD | –22.5 (–29, –17) | –19 (–25, –13) | 0.008 | 0.197 | |  |
| DQD, mean ± SD | 32 (25, 38) | 27.5 (20.8, 34) | 0.002 | 0.226 | |  |

^a^ g/day. ^b^ ml/day. ^c^ Food variety scores ranged from –12 to 0, with lower scores representing less variety of food. Excessive/inadequate/unbalanced dietary intake was evaluated using the high bound score (HBS), low bound score (LBS), and diet quality distance (DQD), respectively. Effect size = Z/√(N) for Mann–Whitney U-tests. ASD, autism spectrum disorder; TD, typically developing; IQR, [interquartile range](javascript:;); SD, standard deviation.

**Table S4. Children with ASD had poorer diets than age-matched TD children (n = 180).**

| **Dietary intake** | **Adjusted β coefficient (95% CI)** | **P-value** | **Effect size** |
| --- | --- | --- | --- |
| Daily vegetable intake^a^ | –46.655 (–88.967 to –4.342) | 0.031 | 0.092 |
| Daily fruit intake^a^ | –33.566 (–66.353 to –0.779) | 0.045 | 0.125 |
| Daily food variety^b^ | –0.828 (–1.608 to –0.047) | 0.038 | 0.142 |
| Inadequate dietary intake^c^ | 3.892 (0.476 to 7.309) | 0.026 | 0.178 |
| Unbalanced dietary intake^c^ | 4.573 (1.069 to 8.076) | 0.011 | 0.215 |

^a^ g/day. ^b^ Food variety scores ranged from –12 to 0, with lower scores representing less variety of food. ^c^ Evaluated using the low bound score (LBS)/diet quality distance (DQD), with higher scores representing a higher degree of inadequate/unbalanced diets. Adjusted for child’s age, sex, intellectual functioning, birth mode, birth order, average daily sleep duration, maternal educational attainment, gestational diabetes mellitus, maternal obesity, monthly per-capita income, and parenting behavior. Effect size = Cohen's f^2^ for linear models. ASD, autism spectrum disorder; TD, typically developing; CI, confidence interval.

**Table S5. Comparisons of GI symptoms between children with ASD and TD children (n = 180).**

| **GI symptoms** | **ASD (n = 90)** | **TD (n = 90)** | **P-value** | **Effect size** |
| --- | --- | --- | --- | --- |
| Constipation, median (IQR) | 0 (0, 1) | 0 (0, 0) | 0.029 | 0.163 |
| Diarrhea, median (IQR) | 0 (0, 0) | 0 (0, 0) | 0.311 | 0.076 |
| Unformed stools, median (IQR) | 0 (0, 0) | 0 (0, 0) | 0.733 | 0.025 |
| Abnormal smell of stool, median (IQR) | 0 (0, 0) | 0 (0, 0) | 0.637 | 0.035 |
| Flatulence, median (IQR) | 0 (0, 0) | 0 (0, 0) | 0.079 | 0.131 |
| Abdominal pain, median (IQR) | 0 (0, 0) | 0 (0, 0) | 0.774 | 0.021 |
| Total GI symptoms scores, median (IQR) | 0 (0, 2) | 0 (0, 1) | 0.093 | 0.125 |

GI symptoms were evaluated by subscale and total scores of the 6-item gastrointestinal severity index (6-GSI). Effect size= Z/√(N) for Mann–Whitney U-tests. GI, Gastrointestinal; ASD, autism spectrum disorder; TD, typically developing; IQR, [interquartile range](javascript:;).

**Table S6. Children with ASD experienced more severe GI symptoms than age-matched TD children (n = 180).**

| **GI symptoms** | **Adjusted β coefficient (95% CI)** | | **P-value** | **Effect size** |
| --- | --- | --- | --- | --- |
| Constipation | | 0.256 (0.039 to 0.472) | 0.021 | 0.086 |
| Total GI symptoms | | 0.474 (0.049 to 0.898) | 0.029 | 0.122 |

GI symptoms include constipation and total GI symptoms. Constipation and total GI symptoms were evaluated by subscale and total scores of the 6-item gastrointestinal severity index (6-GSI). Adjusted for child’s age, sex, intellectual functioning, birth mode, birth order, average daily sleep duration, maternal educational attainment, gestational diabetes mellitus, maternal obesity, monthly per-capita income, and parenting behavior. Effect size = Cohen's f^2^ for linear models. GI, Gastrointestinal; ASD, autism spectrum disorder; TD, typically developing; CI, confidence interval.

**Figure S3. Histogram of the altered gut metabolites correlating with GI symptoms (n = 180). GI symptoms include constipation and total GI symptoms. The data were presented as mean with** [standard deviation](javascript:;) **(SD).** **These significantly altered gut metabolites between children with ASD and TD children** were defined by a fold change **≥ 1.1** or **≤ 0.9,** variable importance in projection (VIP) ≥ 1.25, and P-value < 0.05. GI, gastrointestinal; ASD, autism spectrum disorder; TD, typically developing.
